# Human Neonatal Thymus Mesenchymal Stem Cells Promote Neovascularization and Cardiac Regeneration

**DOI:** 10.1155/2018/8503468

**Published:** 2018-09-16

**Authors:** Shuyun Wang, Shan Huang, Lianghui Gong, Zhize Yuan, Joshua Wong, Jeffrey Lee, Ming-Sing Si

**Affiliations:** Department of Cardiac Surgery, University of Michigan, Ann Arbor, Michigan 48109, USA

## Abstract

Newborns with critical congenital heart disease are at significant risk of developing heart failure later in life. Because treatment options for end-stage heart disease in children are limited, regenerative therapies for these patients would be of significant benefit. During neonatal cardiac surgery, a portion of the thymus is removed and discarded. This discarded thymus tissue is a good source of MSCs that we have previously shown to be proangiogenic and to promote cardiac function in an in vitro model of heart tissue. The purpose of this study was to further evaluate the cardiac regenerative and protective properties of neonatal thymus (nt) MSCs. We found that ntMSCs expressed and secreted the proangiogenic and cardiac regenerative morphogen sonic hedgehog (Shh) in vitro more than patient-matched bone-derived MSCs. We also found that organoid culture of ntMSCs stimulated Shh expression. We then determined that ntMSCs were cytoprotective of neonatal rat cardiomyocytes exposed to H_2_O_2_. Finally, in a rat left coronary ligation model, we found that scaffoldless cell sheet made of ntMSCs applied to the LV epicardium immediately after left coronary ligation improved LV function, increased vascular density, decreased scar size, and decreased cardiomyocyte death four weeks after infarction. We conclude that ntMSCs have cardiac regenerative properties and warrant further consideration as a cell therapy for congenital heart disease patients with heart failure.

## 1. Introduction

Loss of vasculature appears as an important pathophysiological mechanism in heart failure associated with complex congenital heart disease (CHD) [1, 2]. In pressure-overload-induced ventricular failure, myocardial capillary density leads to ischemia and coincides with loss of systolic function [3]. Reduced coronary artery flow caused by morphological abnormalities (such as anomalous coronary artery from the pulmonary artery, coronary artery stenosis, and coronary artery fistula) or from surgical complications (coronary artery obstruction post arterial switch procedure) can also contribute to heart failure in children with CHD [4]. Heart transplantation is the standard treatment for end-stage heart failure in children and is severely limited by the lack of donor organs, limited graft life, and significant side effects of immunosuppressive agents. Therefore, regenerative medicine and cell therapy deserve significant consideration as potential therapies for these children with limited and unsatisfactory treatment options [5, 6].

Open heart surgery in neonates and infants with complex CHD usually requires partial excision of the thymus gland to gain access to the underlying heart and great vessels. We have previously shown that neonatal thymus mesenchymal stem cells (ntMSCs) are proangiogenic and can improve systolic function in a “heart in a dish” model [7, 8]. We found that this improvement was likely due to a paracrine mechanism and that ntMSCs did not differentiate into cardiomyocytes [7].

Sonic hedgehog (Shh) is a secreted factor that has been shown to promote both cardiac regeneration as well as neovascularization in the setting of tissue ischemia [9–11]. The ability of ntMSCs to produce Shh is unknown. Based on these findings, we hypothesized that ntMSCs may provide protective and regenerative effects in the setting of myocardial ischemia. To evaluate this hypothesis, we performed experiments to determine if ntMSCs could express Shh, be cytoprotective to cardiomyocytes in vitro, and promote cardiac regeneration in vivo.

## 2. Materials and Methods

### 2.1. Cell Isolation and Culture

All studies were performed under an approved protocol from the University of Michigan Institutional Review Board. The isolation, detailed characterization, and culture of neonatal thymus (nt) MSC and bone (nb) MSC lines used in this study have been described previously [8, 12]. Briefly, after informed consent was given by the parents, discarded thymus and sternal bone tissue from infant heart operations were mechanically minced into <3 mm fragments under sterile conditions. Tissue fragments were placed in 100 mm culture dishes and submerged in MSC media (Dulbecco's modified Eagle medium, with high-glucose concentration, GLUTAMAX I, 10% heat inactivated fetal bovine serum, 100 U/mL penicillin, and 100 *μ*g/mL streptomycin, all from Gibco, Carlsbad, CA). Tissue fragments were incubated for 10–14 days prior to removal. MSCs that had migrated from the tissue explants were allowed to achieve 80% confluence prior to passaging with trypsin/EDTA (Gibco). Human umbilical vein endothelial cells (HUVECs) were cultured in EGM-2 (both from Lonza, Basel, Switzerland) with growth factors under standard conditions, unless stated otherwise. Unless specified otherwise, all experiments were performed with cells from passages 3–9.

### 2.2. Hanging Drop Culture

HUVEC, nbMSC, and ntMSC cell cultures were dissociated using trypsin/EDTA, centrifuged, and resuspended in EGM-2 media containing 0.275% methylcellulose (Sigma). Cells in suspension were then seeded on the lid of a nonadhesive Petri dish (20 *μ*L per drop) containing 800 cells/hanging drop. For HUVEC + MSC combinations, 400 cells from each cell type was used to make the hanging drop. Hanging drops were then incubated overnight at 37°C and 5% CO_2_ to allow for spheroid formation.

### 2.3. Gene Expression Analysis

Differential gene expression in the different cell types and culture conditions were determined with qPCR. Total RNA was extracted from cells, hanging drops, or cell sheets using the RNeasy Mini Kit (Qiagen, Valencia, CA). Reverse transcription was carried out using the High Capacity cDNA Reverse Transcription Kit with random primers (Applied Biosystems, Invitrogen). Quantitative real-time polymerase chain reaction was performed in StepOne Plus Real time PCR System (Applied Biosystems) with a reaction mixture (10 *μ*L) containing cDNA, forward and reverse primers (see below), and 1x iTaq Universal SYBR Green Supermix (Bio-Rad Laboratories, Hercules, CA). Expression of each gene was normalized to the expression of *β*-actin. Mean cycle threshold (Ct) value was calculated as the average of triplicates for each gene, and the fold change in gene expression was calculated based on 2-ΔΔCT method. Primers used in this study are listed in Table 1.

### 2.4. Shh ELISA

Shh levels in conditioned media from monolayer or hanging drop culture were measured using an ELISA kit (RayBiotech, Norcross, GA) according to the manufacturer's directions. Conditioned media from monolayers were generated by incubating 5 × 10^5^ cells in flasks with serum-free DMEM for 5 days. Conditioned media from hanging drops were generated by collecting culture supernatants from hanging drops using centrifugation. When comparing conditioned media from monolayers and hanging drops, the same number of cells and volume of supernatant were used. Two independent experiments were performed and conditioned media samples were tested in triplicate by ELISA.

### 2.5. H_2_O_2_ Oxidative Stress Assay

Neonatal rat cardiomyocytes (CMs) were isolated as previously described [7]. CMs were then cultured in 24 well plates (5 × 10^4^ CMs/well) for 24 hours using MSC media and standard culture conditions. Then, H_2_O_2_ was added to achieve a final concentration of 50 *μ*m. In one group, 5 × 10^4^ ntMSCs were seeded onto porous cell culture inserts which were then inserted into wells containing CMs treated with H_2_O_2_. In another group, 5 × 10^4^ ntMSCs were added to the well containing CMs treated with H_2_O_2_ to permit direct contact. After 4 hours, cells were harvested for apoptosis analysis by flow cytometry and TUNEL staining.

Apoptosis was detected using a fluorochrome conjugated anti annexin V antibody (BD Biosciences, San Jose, CA). The antibody was incubated with cells for 60 minutes at room temperature followed by three washes. Stained cells were then analyzed using a MoFlo® Astrios™ flow cytometer (Beckman Coulter Inc.) using the appropriate isotype-matched and unstained controls. TUNEL staining was performed using the In Situ Cell Death Detection Kit (Sigma-Aldrich Corp., St. Louis, MO) according to the manufacturer's protocol. Briefly, cells were fixed with freshly prepared 4% PFA in PBS for 1 hour at room temperature, then permeabilized with 0.1% Triton X 100 in 0.1% sodium citrate for 2 minutes on ice. TUNEL reaction mixture was added and incubated in the dark at 37°C for 1 hour in a humidified incubator. The TUNEL positive cells were quantified in 5 randomly chosen high power fields.

### 2.6. Scaffoldless ntMSC Cell Sheet Generation

Scaffoldless ntMSC cell sheets were generated by culturing 4 × 10^6^ ntMSCs in thermoresponsive polymer coated 35 mm culture dishes (Nunc™ Dishes with UpCell™ Surface, Thermo Fisher Scientific, Waltham, MA) for 48 hours under standard culture conditions. Cell sheets lifted off the culture surface and were harvested for use after exposing the dishes to room temperature for 10 minutes.

### 2.7. Rat Coronary Ligation Model

The care of animals was in accordance with institutional guidelines. Male RNU nude rats (*n* = 13, 200–250 grams, Charles River, Wilmington, MA) underwent left coronary artery ligation after endotracheal intubation, mechanical ventilation, and left thoracotomy. ntMSC cell sheets were placed on the epicardial surface of the left ventricle (LV) in 8 randomly chosen animals. On days 3 and 28, all animals underwent a sedated echocardiogram (VisualSonics Vevo 770, FUJIFILM VisualSonics Inc., Toronto, ON, Canada) to determine ejection fraction and fractional shortening from the parasternal short axis view [13]. After the last echocardiogram, animals were euthanized and the hearts were harvested after administration of a potassium rich solution to ensure diastolic arrest. Heart tissue was then formalin fixed, paraffin embedded, sectioned at 5 *μ*m thickness, and stained with H&E and Masson's Trichrome according to routine histology protocols. The infarct size was analyzed using Image J software. For immunohistochemistry studies, heart sections were deparaffinized and rehydrated. After antigen retrieval, the slides were treated differently according to the further staining purposes. For vWF staining, the sections were incubated in 0.3% peroxide hydrogen for 30 min to block the endogenous peroxidase activity. Then, the sections were incubated with rabbit polyclonal vWF primary antibody (anti-von Willebrand factor antibody, ab6994, Abcam) overnight at 4°C. Following three washes with PBS, the sections were incubated with biotinylated goat anti-rabbit immunoglobulin G and a streptavidin-biotin complex, respectively. Then, they were incubated with diaminobenzidine (DAB) for 3 min at room temperature, counterstained with hematoxylin, cleared, mounted, and examined. For immunofluorescence staining, the specimens were blocked with 10% goat serum and proceeded overnight primary antibody (mouse monoclonal anti-*α*-actinin, clone EA-53, Sigma-Aldrich) incubation at 4°C. Secondary antibody was goat anti-rabbit Alexa Fluor 488 (Invitrogen, Eugene, OR). Sections were then stained with DAPI. TUNEL staining was performed using the In Situ Cell Death Detection Kit (Sigma-Aldrich Corp., St. Louis, MO) according to the manufacturer instructions, as described above.

### 2.8. Statistical Analysis

Data were expressed as mean ± SD. When subject-matched comparisons were performed, a paired *t*-test was used. Statistical differences between multiple groups were determined using one-way analysis of variance (ANOVA) with post hoc Tukey's Honestly Significant Difference test. Significance was defined as a *p* value < 0.05.

## 3. Results and Discussion

**Figure 1:**
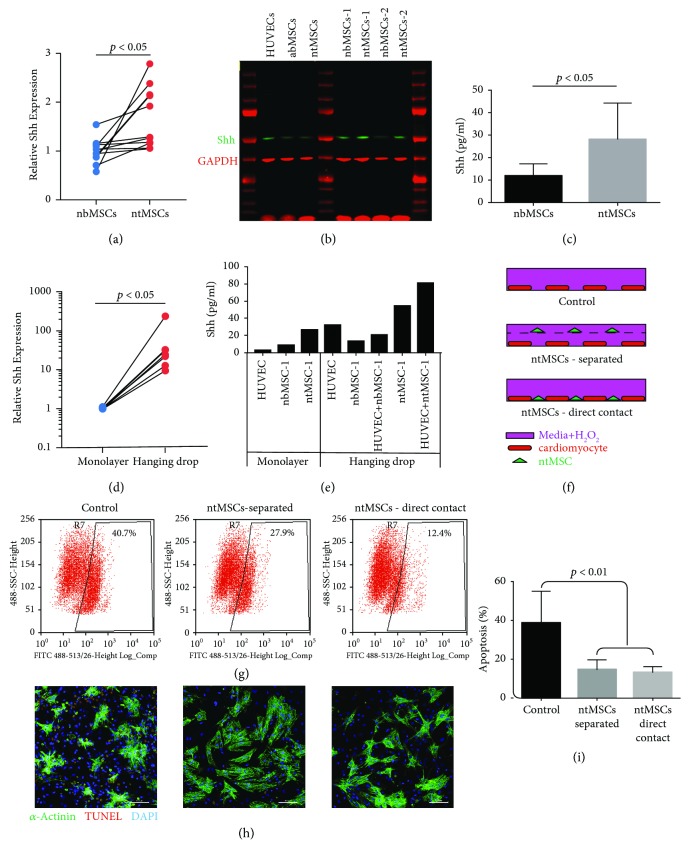
(a) Subject-matched ntMSCs possess a higher Shh expression as compared to nbMSCs. All cells were cultured in monolayers prior to analysis. Comparison performed with a paired *t*-test. (b) Shh cellular protein levels are higher in ntMSCs as compared to adult bone (a, b) MSCs and subject-matched nbMSCs (numbers denote subject). As another comparison, Shh content was also measured in monolayer-cultured HUVECs. (c) Conditioned media from ntMSCs contained a higher concentration of Shh than that from subject-matched nbMSCs as determined by ELISA. Mean ± SD is shown of three technical replicates. Data is representative of two independent experiments performed with MSCs isolated from two subjects. (d) Hanging drop culture of ntMSCs stimulated Shh expression. Data are from ntMSCs from five subjects and comparison was performed with a paired *t*-test. (e) Hanging drop culture stimulated Shh secretion. Conditioned media was generated from HUVECs and subject-matched nbMSCs and ntMSCs cultured in monolayers of hanging drops. Hanging drop culture of HUVEC + ntMSCs stimulated the most Shh secretion. (f) Experimental setup to determine the cytoprotective effects of ntMSCs on H_2_O_2_-induced death of neonatal rat CMs. Controls were exposed to H_2_O_2_ in the absence of ntMSCs. The second group contained ntMSCs cultured on an insert, thereby sharing the same media but was physically separated from the CMs. The third group containing ntMSCs cocultured with CMs on the same culture surface permitted direct contact between the two cell types. (g) H_2_O_2_ induced apoptosis in all groups but was decreased in the presence of ntMSCs. The most cytoprotection was seen when ntMSCs were cultured in direct contact with the CMs. Results are representative of two independent experiments. (h, i) TUNEL staining confirmed that the presence of ntMSCs prevented apoptosis. Results are representative of two independent experiments. Scale bar = 100 *μ*m (h).

We first determined if ntMSCs expressed and secreted Shh in vitro. Shh transcript expression was determined by qPCR in pairs of ntMSCs and neonatal sternal bone (nb) MSCs isolated from 7 patients (Figure 1(a)). We found that ntMSCs expressed Shh at significantly increased levels as compared to nbMSCs (Figure 1(a)). We then confirmed these findings with Western blot (Figure 1(b)) and ELISA of conditioned media from nbMSCs and ntMSCs (Figure 1(c)). Because our prior results indicated that high density, 3D culture of MSCs could regulate gene expression [8], we next determined if spheroid formation by hanging drop culture of ntMSCs would stimulate Shh expression. We found that hanging drop culture significantly stimulated the transcription of Shh in ntMSCs isolated from different subjects (Figure 1(d)). The amount of secreted Shh was also quantified by ELISA and confirmed that organoid culture of ntMSCs stimulated Shh secretion (Figure 1(e)). Collectively, these results indicated that ntMSCs express and secrete the Shh and that this is stimulated by high density, 3D culture.

We next asked if ntMSCs could exert cytoprotective effects on cardiomyocytes in the setting of oxidative stress, as this type of injury is present in the setting of myocardial ischemia and heart failure [14, 15]. We exposed neonatal rat cardiomyocytes (CMs) to H_2_O_2_ and determined if the presence of ntMSCs (either separated but sharing same media or in direct contact) influenced CM survival (Figure 1(f)). We found that H_2_O_2_-induced CM death was abrogated by ntMSCs as determined by Annexin V surface expression (Figure 1(g)) and TUNEL staining, and the greatest amount of cytoprotection was noted when ntMSCs were in direct contact with CMs (Figures 1(h) and 1(i)).

**Figure 2:**
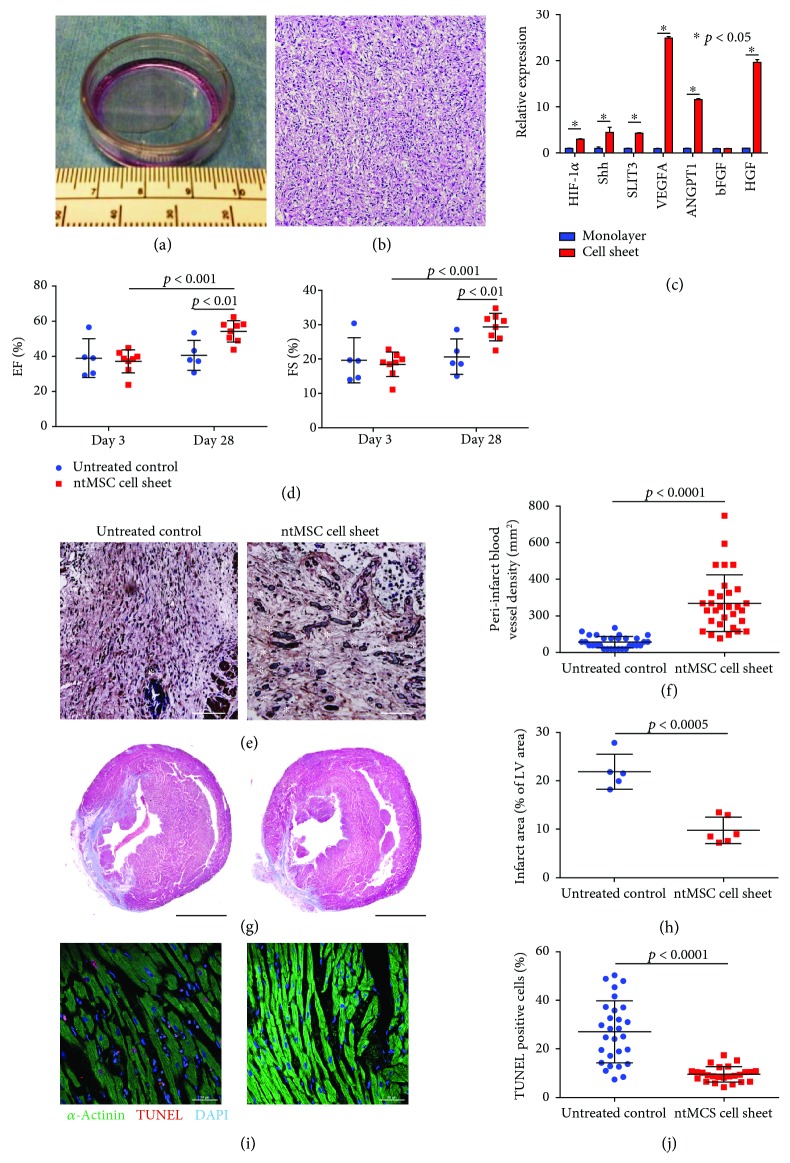
(a) Scaffoldless cell sheet made by culturing 4 × 10^6^ ntMSCs in a thermoresponsive polymer-coated 35 mm dish. (b) H&E stain of an ntMSC cell sheet demonstrates high cell density, 3D organization. Scale bar = 100 *μ*m. (c) Cell sheet generation stimulated the expression of proangiogenic genes in ntMSCs as compared to monolayer culture. (d) Echocardiogram evaluation of untreated controls (*n* = 5) and ntMSC cell sheet-treated RNU nude rats (*n* = 8) that underwent left coronary ligation. Ejection fraction (EF) and fractional shortening (FS) for both groups were identical 3 days after left coronary ligation but only the ntMSC cell sheet treated animals showed significant improvement at 28 days. (e, f) Immunohistochemistry with anti-vWF antibody demonstrated that the vascular density in the peri-infarct region increased with ntMSC cell sheet treatment. White asterisks denote blood vessel lumens containing red blood cells. Scale bars = 50 *μ*m. (g, h) Masson trichrome stain demonstrated that the postinfarct scar region (blue) was decreased by ntMSC cell sheet treatment. Scale bars = 3 mm. (i, (j) Confocal microscopy and TUNEL staining revealed decreased apoptosis in the LV myocardium of ntMSC cell sheet-treated animals. Scale bars = 50 *μ*m.

In light of these findings, we then asked if ntMSCs could mitigate the effects of severe myocardial ischemia in a rat model of myocardial infarction achieved by left anterior descending coronary artery ligation. Since our prior [8] and above results (Figure 1) indicated that high density, 3D culture of ntMSCs activated the expression of Shh and other proangiogenic factors, we decided to deliver the ntMSCs to the ischemic regions of the left ventricle in the form of scaffoldless cell sheets (4 × 10^6^ ntMSCs/sheet) formed by culturing on thermoresponsive polymer-coated culture surfaces (Figure 2(a)) [16] and resulted in a high cell density, 3D arrangement (Figure 2(b)). Furthermore, cell sheet creation stimulated the expression of Shh and other proangiogenic genes as compared to monolayer culture of ntMSCs (Figure 2(c)).

Immediately after left coronary ligation, we placed ntMSC cell sheets onto the surface of the left ventricle in the treatment group (*n* = 8 animals), whereas control animals did not receive cell sheets (*n* = 5 animals). Echocardiogram at day 3 revealed that LV ejection fraction (EF) and fractional shortening (FS) were significantly depressed in all animals (Figure 2(d)). At 4 weeks, the animals underwent a second echocardiogram which demonstrated that untreated controls (*n* = 5) did not demonstrate any change in LV function over the study period whereas treatment animals (*n* = 8) demonstrated a significant improvement in LV ejection fraction (Figure 2(d)). Furthermore, there was increased peri-infarct vWF + blood vessel density in the treated group (Figures 2(e) and 2(f)) and decreased scar size (Figure 2(g) and Figure 2(h)). We then evaluated the amount of cell death in the LV in both groups by using TUNEL staining. We found that ntMSC cell sheet treatment significantly decreased the frequency of TUNEL positive-stained CMs, indicating a cytoprotective effect (Figures 2(i) and 2(j)).

Our results indicate that discarded thymus tissue obtained from neonatal cardiac surgery are a source of MSCs that possess regenerative properties. These thymus-derived MSCs are able to secrete Shh and to protect CMs against cytotoxic oxidative stress in vitro, and further work is needed to determine how much of these properties contribute to the therapeutic effects seen in vivo. A distinguishing characteristic of ntMSCs is its higher expression and secretion of Shh as compared to bone-derived MSCs. Jia et al. forced the expression of Shh in bone-derived MSCs to increase their therapeutic potency in a spinal cord injury model [17]. Our results suggest that Shh-endowed, nonmodified ntMSCs may also have therapeutic utility in this model and clinical situation.

While MSCs from multiple tissue types have demonstrated salutatory cardiac properties in the preclinical settings, results of clinical trials have been overall disappointing [18, 19]. Therefore, rigorous and unbiased head-to-head comparisons are needed to determine which tissue source provides the MSC with the greatest therapeutic potency.

## 4. Conclusions

In the neonate with critical CHD undergoing cardiac surgery who is also at significant risk for developing heart failure later in life, the discarded thymus tissue is a practical and ample source of cardiac regenerative MSCs that can be expanded and cryopreserved, and thus ntMSCs warrant further evaluation for clinical translation.

## Figures and Tables

**Table 1:** 

Gene	Forward primer	Reverse primer
*β*-*Actin*	TCCCTGGAGAAGAGCTACGA	AGCACTGTGTTGGCGTACAG
*Shh*	GATGTCTGCTGCTAGTCCTCG	CACCTCTGAGTCATCAGCCTG
*Slit3*	TGATGGCAACGAGGAGAGTA	ACGGCTGTTAGGTGGTTTCC
*HGF*	CTCACACCCGCTGGGAGTAC	TCCTTGACCTTGGATGCATTC
*HIF-1α*	CAGCAACTTGAGGAAGTACC	CAGGGTCAGCACTACTTCG
*VEGFA*	GCCTTGCTGCTCTACCTCCA	ATGATTCTGCCCTCCTCCTTCT
*bFGF*	GCTGGTGATGGGAGTTGTATTT	CTGCCGCCTAAAGCCATATT
*ANGPT1*	GCTCACCATCATCTCCCTTATC	CTCACAGACTCAATCACCTTCC

## Data Availability

The qPCR, ELISA, echocardiogram, histology, and immunohistochemistry data used to support the findings of this study are available from the corresponding author upon request.
